# Cognitive Frailty and Its Long-Term Effect on Depression in an Older Population in Korea

**DOI:** 10.1093/geroni/igab046.1708

**Published:** 2021-12-17

**Authors:** Yoonjung Ji, TaeWha Lee, Eunkyung Kim

**Affiliations:** Yonsei University, Seoul, Seoul-t'ukpyolsi, Republic of Korea

## Abstract

Cognitive frailty is a condition where physical frailty and mild cognitive impairment (MCI) co-exist without dementia. It occurs in 1.8%-8.9% of the general older population, and older people with depression have a higher risk of frailty. However, the relationship between cognitive frailty and depression is still unclear. This study aimed to determine the relationship between cognitive frailty and depression of older adults by time using comparative group analysis. A secondary analysis was conducted using the Korean Longitudinal Study of Aging (KLoSA) dataset from 2010 to 2018. A sample was 981 older adults who were 65 years old and without dementia over residing in the community. Cognitive frailty was defined as having a mini-mental state examination score of 18-23 and 3 or more of the Fried frailty indexes. Generalized Estimating Equation model and chi-square test were employed. Of the 981 subjects, the cognitive frailty(CF) was 28.5%, followed by robust (37.7%), physical frailty (PF, 29.4%), mild cognitive impairment (MCI, 4.4%) at baseline. The group differences on depression measured by the Center for Epidemiological Studies Depression (CESD) were statistically significant in the PF (F=4.70, p<.001) and the CF (F=4.95, p<.001) group compared to the robust group. The time difference effect (F=.09, p=.05) and a group-by-time interaction effect were observed (p<.001). This study confirmed that cognitive frailty is strongly associated with depression. Effective approaches to managing psychological wellbeing, including dementia, are essential for older adults with cognitive frailty.

